# Dietary Copper Reduces the Hepatotoxicity of (−)-Epigallocatechin-3-Gallate in Mice

**DOI:** 10.3390/molecules23010038

**Published:** 2017-12-23

**Authors:** Najeeb Ahmed Kaleri, Kang Sun, Le Wang, Jin Li, Wenzheng Zhang, Xuan Chen, Xinghui Li

**Affiliations:** 1 Tea Research Institute, Nanjing Agricultural University, Nanjing 210095, China; 2014204039@njau.edu.cn (N.A.K.); sunkang@njau.edu.cn (K.S.); 2014104091@njau.edu.cn (L.W.); 2015204026@njau.edu.cn (J.L.); 2 Department of Regenerative and Cancer Cell Biology, Albany Medical College, Albany, NY 12208, USA; zhangw1@mail.amc.edu

**Keywords:** tea, (−)-epigallocatehin-3-gallate, copper, ceruloplasmin, hepatotoxicity

## Abstract

We developed Cu-deficient, -sufficient and -super nutrition mice models by feeding them with diet containing 1.68, 11.72 or 51.69 mg of Cu/kg for 28 days, respectively. Then, the mice were treated to (−)-epigallocatechin-3-gallate (EGCG, 750 mg/kg BW) by oral in order to assess the acute toxicity of the drug. Following EGCG treatment, the survival rates were 12.5%, 50% and 100% in the Cu-deficient, -sufficient and Cu-super nutrition groups of mice, respectively. Cu level and ceruloplasmin activity in serum were significantly increased with the increase of dietary Cu. However, the Cu supplementation did not produce any obvious impact on serum superoxide dismutase activity. Furthermore, ceruloplasmin, in vitro, significantly promotes EGCG oxidation accompanied with increasing oxidation products and decreasing levels of reactive oxygen species. These results, therefore, suggest that Cu can relieve EGCG hepatotoxicity, possibly by up-regulating ceruloplasmin activity, which can be used to promote EGCG applications.

## 1. Introduction

Tea (*Camellia sinensis*), prepared from the leaves of tea plant, is one of the most important and famous beverages consumed all over the world. Green tea, a rich source of a class of flavonoid compounds referred to as catechins [1], possess many beneficial effects on human health, including antioxidant, anti-inflammatory, anticancer, and antibacterial effects [2,3,4].

Green tea catechins were widely studied because of their potential benefits on human health [5]. (−)-Epigallocatechin-3-gallate (EGCG), the predominant component among catechins, can prevent certain types of chronic diseases including cancer, obesity, type-2 diabetes, lipid metabolism abnormity, atherosclerosis, and other cardiovascular diseases [6]. Intriguingly, EGCG can also undergo auto-oxidation to generate reactive oxygen species (ROS). At physiological dose, the moderate pro-oxidant action of EGCG induces adaptive responses with the gain of enhanced and prolonged antioxidant and detoxifying capacities, which has been regarded as an important mechanism for many of the beneficial biological functions of EGCG [7].

However, many researchers have reported that high doses of green tea extract or EGCG can induce hepatotoxicity. A study has revealed that high dose of EGCG caused hepatotoxicity and even death in mice [8]. The oral administration of EGCG at dose of 2000 mg/kg induced 80% mortality [9]. Green tea polyphenols, containing 90% EGCG, induced toxicity and even death in Beagle dogs [10]. Consumption of the green tea extract at high doses (expressed as EGCG 270 and 400 mg/day) caused hepatotoxicity in human beings [11,12,13]. Moreover, green tea hepatotoxicity is greatly enhanced when it is consumed in association with other herbal or dietary ingredients [13]. However, the melatonin attenuated EGCG-triggered hepatotoxicity and reduced hepatic Nrf2 activation [12], suggesting that there exist some factors that can affect EGCG hepatotoxicity.

Copper (Cu) is an essential metal for mammals, which is readily absorbed across the small intestine. The liver represents the predominant storage organ for Cu and the excess Cu is excreted in bile and eliminated in feces. Many enzymes require stoichiometric amounts of Cu to catalyze key reactions [14]. For example, copper/zinc superoxide dismutase (SOD) 1 is an important cuproenzyme. This enzyme requires Cu for full catalytic activity to catalyze the dismutation of superoxide into molecular oxygen and hydrogen peroxide. Ceruloplasmin (Cp) is a multicopper oxidase and the most abundant Cu binding protein in blood, which contains 95% of the Cu found in serum [15]. The main function of Cp is to participate in iron homeostasis due to its ferroxidase activity by converting ferrous to ferric iron. Mice developed anemia with low tissue Cu levels in liver or kidney and decreased Cp activity, but not ferroxidase activity in serum [14]. However, excessive Cu causes DNA damage, lipid peroxidation and protein dysfunction because of its exceptionally high redox activity [16].

The auto-oxidation of EGCG results in generation of ROS. It is principally mediated by superoxide anions or Cu ion and is inhibited by SOD in vitro [17,18]. Dietary Cu can have an impact on Cu levels, Cp, and SOD1 in tissues [14,19,20]. Although, several literatures reported that Cu levels in tissues can enhance the capacity of flavonoids on cancer cell toxicity and DNA damage in vitro [21,22,23,24,25,26], the effects of dietary Cu on EGCG toxicity have not been examined. In this study, we intended to address this question, using mice fed with diets containing various levels of Cu as the model systems.

## 2. Results

### 2.1. Generation of Mice Models with Different Copper Status

**Figure 1:**
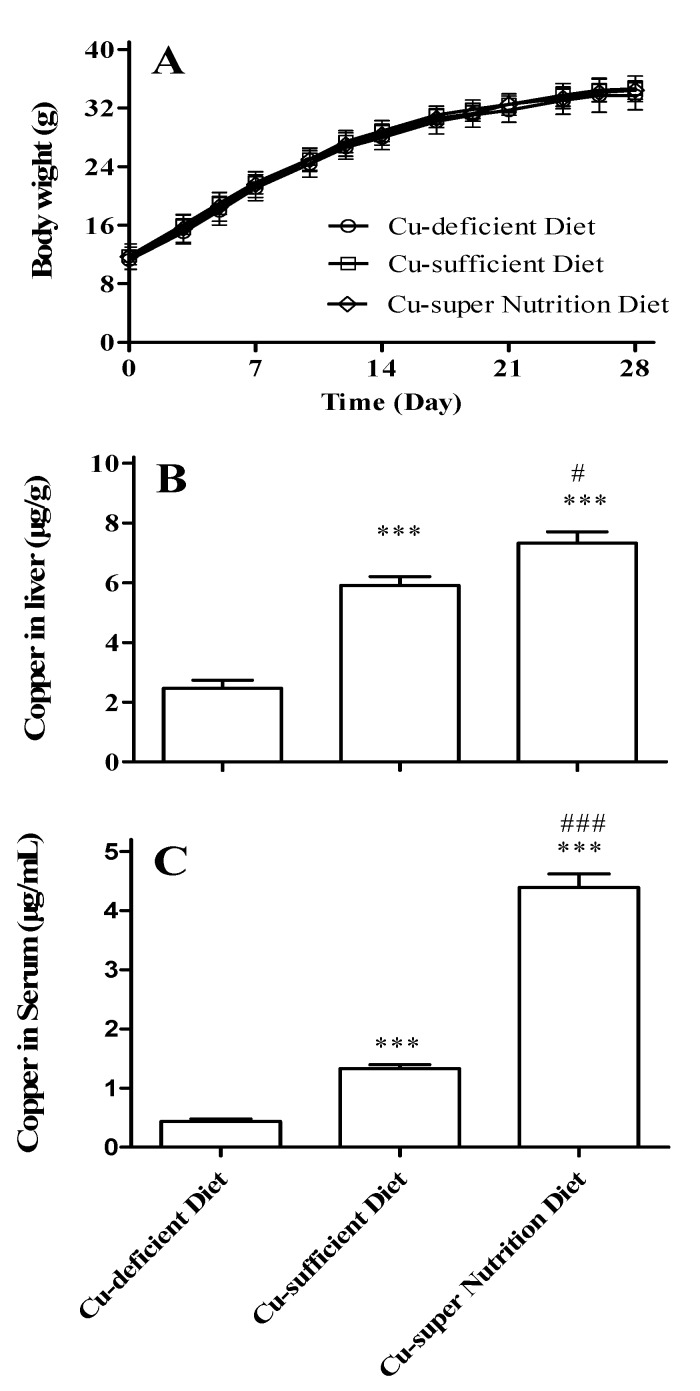
Effect of copper diets on body weight and copper levels in mice. The effect of Cu diet levels on the body weight (**A**); Cu levels in liver (**B**) and serum (**C**) of mice, which were feed with diets containing different Cu contents (1.68, 11.72 or 51.69 mg/kg) for 28 consecutive days. All data were presented as mean ± SEM (at least *n* = 6). Compared to Cu-deficient diet, *** *p* < 0.001; compared to Cu-sufficient diet, # *p* < 0.05; ### *p* < 0.001.

The body weights were statistically indistinguishable between any models (Figure 1A), suggesting that the Cu diet treatments did not affect the overall growth of mice. Nevertheless, we found that the liver Cu levels of the three models were 2.46 ± 0.27, 5.91 ± 0.30 and 7.32 ± 0.39 μg/g, respectively (Figure 1B, all *p* < 0.05), and the liver Cu levels of the three models were 0.44 ± 0.04, 1.33 ± 0.07 and 4.39 ± 0.23 μg/mL respectively (Figure 1C, all *p* < 0.001). These results indicate that the different Cu levels mice models were indeed established.

### 2.2. Copper Decreased the Lethal Effects of EGCG

**Figure 2:**
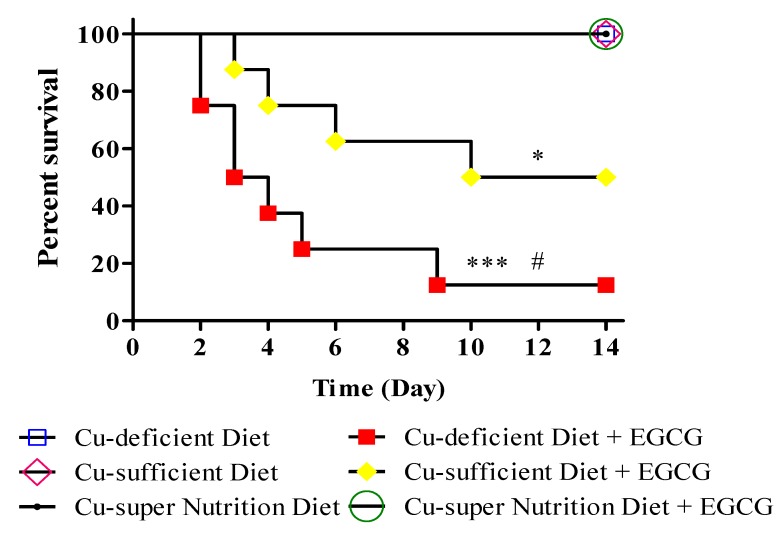
Survival curve after (−)-Epigallocatechin-3-gallate (EGCG) treatments in copper diet-feed mice. Mice (*n* = 8) were fed with diets with different Cu contents (1.68, 11.72 or 51.69 mg/kg) for 28 consecutive days. Then, mice were i.g. injected with a single dose of EGCG (750 mg/kg). Compared to control group, * *p* < 0.05, *** *p* < 0.001; compared to Cu-sufficient diet plus EGCG, # *p* < 0.05.

The impact of Cu levels on EGCG-induced mortality; the remaining sixteen mice were monitored for 14 days after saline or a single dose of EGCG (750 mg/kg by oral) treatment, respectively. Although EGCG at tested dose did not cause death in mice fed with Cu-super nutrition, it induced severe toxicity resulting in death beginning at day 2 in Cu-deficient and at day 3 in Cu-sufficient mice. Fourteen days after EGCG treatment, the survival rates were 12.5%, 50% and 100% in Cu-deficient, -sufficient, and -super nutrition models, respectively (Figure 2). All mice, regardless of their Cu status, survived the saline administration. These results demonstrated that Cu can significantly decrease the EGCG-induced mortality in mice.

### 2.3. Copper Reduced the Acute Hepatotoxicity of EGCG

**Figure 3:**
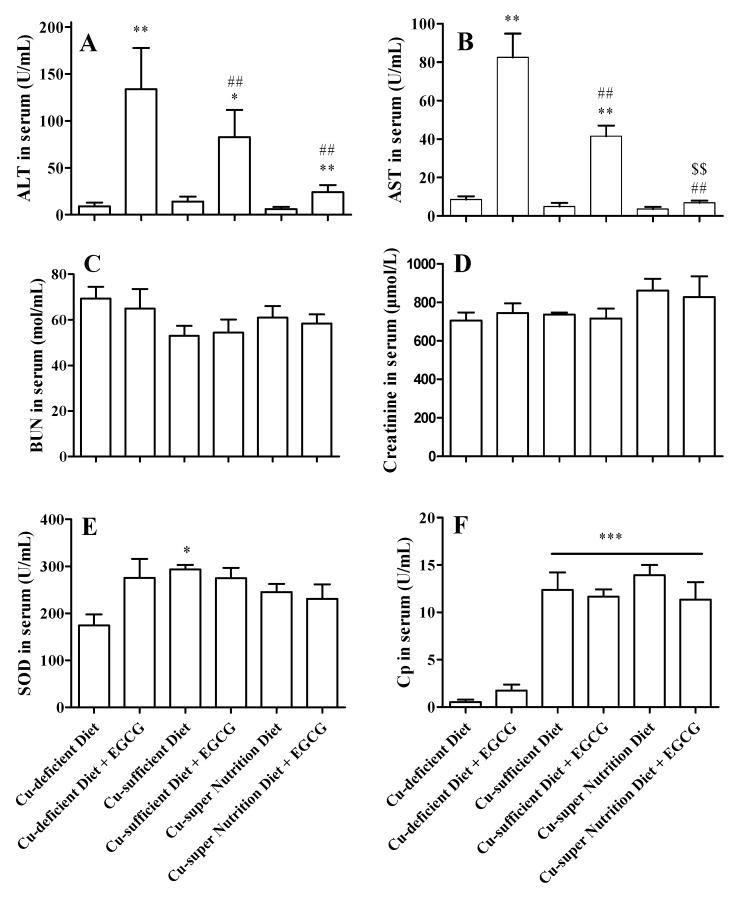
Effect of EGCG on serum biomarkers in copper diet-feed mice. Mice were fed with diets with different Cu contents (1.68, 11.72 or 51.69 mg/kg) for 28 consecutive days. Then, mice were i.g. injected with a single dose of EGCG (750 mg/kg), and then were sacrificed at 24 h after EGCG group. Activities of ALT (**A**); AST (**B**); BUN (**C**); Creatinine (**D**); SOD (**E**); and Cp (**F**) in serum were detected. All data were presented as mean ± SEM (*n* = 6). Compared to Cu-deficient diet, * *p* < 0.05, ** *p* < 0.01, *** *p* < 0.001; compared to Cu-deficient diet plus EGCG, ## *p* < 0.01; compared to Cu-sufficient diet plus EGCG, $$ *p* < 0.01.

To investigate the effect of Cu on the acute toxicity of EGCG, fourteen mice per Cu level model were randomly divided into two equal subgroups. The mice were administered with saline as control and EGCG at a dose of 750 mg/kg by gavage, and then, were sacrificed 1 day later. Serum ALT and AST activities were significantly increased after EGCG treatments, and reversely correlated with the dietary Cu concentration (Figure 3A,B), demonstrating a beneficial effect of dietary Cu in relieving the EGCG-induced acute liver damage. The levels of BUN, creatinine and SOD in serum were not significantly changed after the drug administration (Figure 3C–E) in each of the Cu status models, implying that there was no severe acute kidney injury caused by the EGCG treatment. Serum Cp is a sensitive biomarker for Cu deficiency [19]. The dietary Cu induced a dose-dependent effect on Cp. Compared to Cu-deficient model, Cu-sufficient and Cu-super nutrition models possessed significantly elevated serum Cp activities when they were treated with either saline or EGCG (Figure 3F).

**Figure 4:**
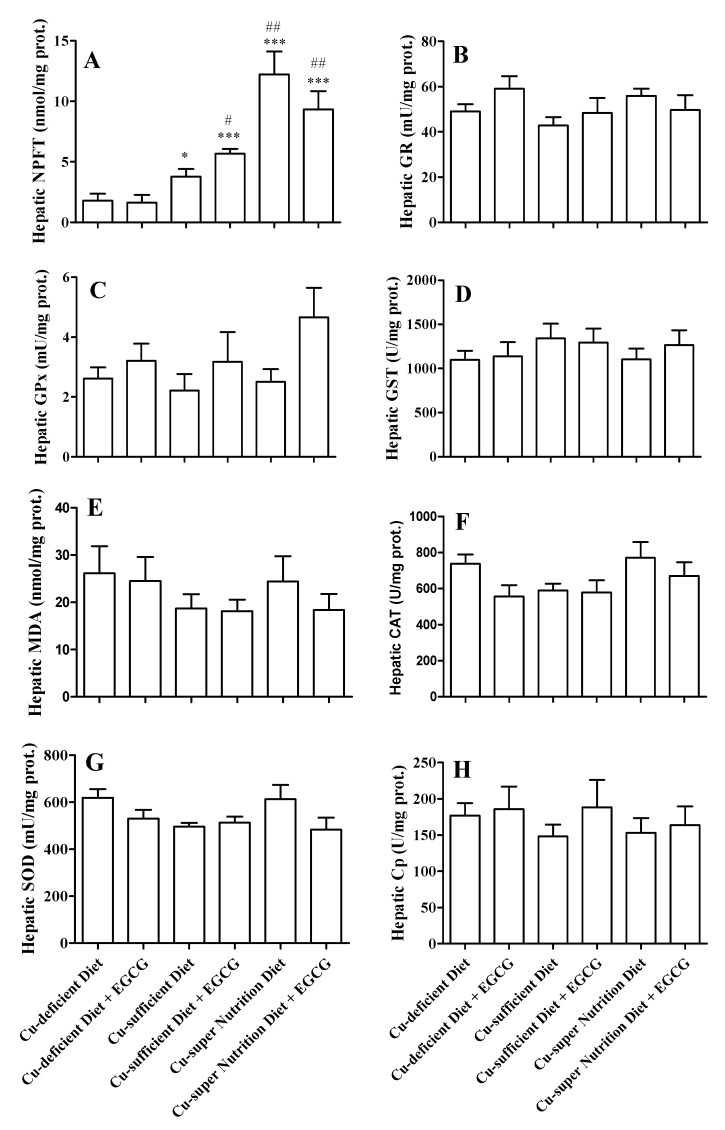
Effect of EGCG on activities on anti-oxidant enzymes and copper proteins in copper diet-feed mice. Mice were fed with diets with different Cu contents (1.68, 11.72 or 51.69 mg/kg) for 28 consecutive days. Then, mice were i.g. injected with a single dose of EGCG (750 mg/kg), and then were sacrificed at 24 h after EGCG group. Hapetic GSH (**A**); GR (**B**); GPx (**C**); GST (**D**); MDA (**E**); CAT (**F**); SOD (**G**) and Cp were measured. All data were presented as mean ± SEM (*n* = 6). Compared to Cu-deficient diet, * *p* < 0.05, *** *p* < 0.001; compared to Cu-sufficient diet, # *p* < 0.05, ## *p* < 0.01.

To further investigate the beneficial effects of dietary Cu, we measured the activities of several antioxidant enzymes in liver. Dietary Cu significantly increased NPFT levels, apparently in a dose-dependent manner. For saline-treated mice, NPFT level was significantly increased from 1.80 ± 0.57 nmol mg^−1^ protein in the Cu-deficient model to 3.79 ± 0.64 and 12.22 ± 1.91 nmol mg^−1^ protein in Cu-sufficient and -super nutrition models, respectively (Figure 4A, all *P* < 0.05). A similar pattern of changes in NPFT levels was also observed in the EGCG-treated subgroups (Figure 4A). However, the activities of other antioxidant enzymes including GR, GPx, GST, MDA and CAT in liver were not altered by dietary Cu and EGCG administration (Figure 4B–F).

**Figure 5:**
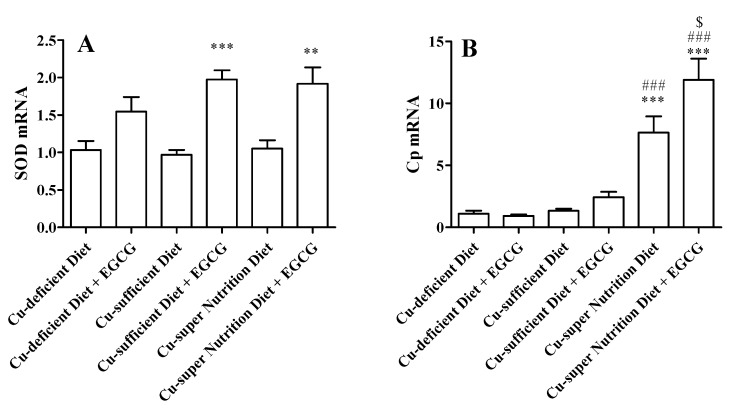
Impact of EGCG on the hepatic superoxide dismutase (SOD) and Cp mRNA levels in Cu diet-feed mice. Mice were fed with diets with different Cu contents (1.68, 11.72 or 51.69 mg/kg) for 28 consecutive days. Then, mice were i.g. injected with a single dose of EGCG (750 mg/kg), and then were sacrificed at 24 h after EGCG group. (**A**) SOD mRNA; (**B**) Cp mRNA. Data are presented as mean ± SEM (*n* = 6). Compared to Cu-deficient diet, ** *p* < 0.01, *** *p* < 0.001; compared to Cu-sufficient diet, ### *p* < 0.001; compared to Cu-super nutrition diet, $ *p* < 0.05.

Although hepatic Cp activities were not significantly changed after the drug administration (Figure 4H), consistent with the elevated serum Cp activities, qRT-PCR analyses revealed that the liver Cp mRNA was 7.6- and 11.9-fold higher in EGCG-treated Cu-sufficient and -super nutrition models, compared to EGCG-treated Cu-deficient mice (Figure 5B). SOD is an important Cu enzyme that scavenges superoxide anions inhibiting the auto-oxidation action of EGCG. We found that SOD activities in serum and liver were not significantly altered by the EGCG treatment in each of the three models (Figure 3E and Figure 4G). Nevertheless, the Cu-super nutrition vs. Cu-deficient mice had a 2-fold increase in SOD mRNA expression in the liver (Figure 5A).

### 2.4. Cp Promotes EGCG Oxidation with Low Generation of ROS In Vitro

**Figure 6:**
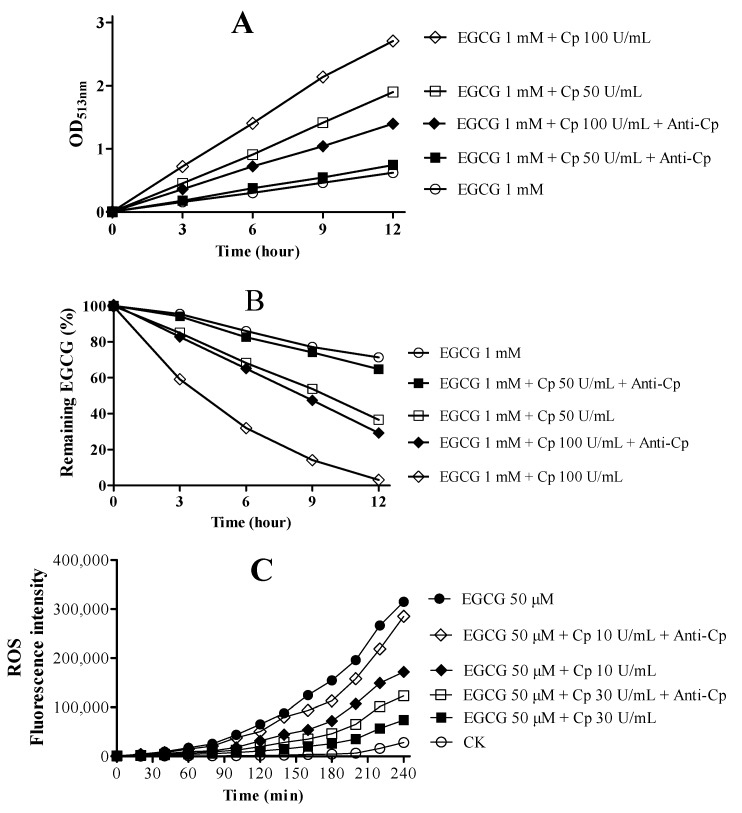
Impact of Cp on EGCG oxidation. (**A**) EGCG oxidation products detected by OD_513nm_; (**B**) Stability of EGCG evaluated by HPLC. (d) ROS detected by DCFH-DA fluorescence. Chemicals were mixed in 0.15 M PBS (pH 7.4) incubated at 25 °C for 12 h in (**A**,**B**), 0.15 M PBS (pH 7.4) incubated at 37 °C for 4 h in (**C**). Anti-Cp concentrations were 10 μg/mL in (**A**,**B**) and 5 μg/mL in (**C**). Data are presented as mean ± SEM (*n* = 3).

To investigate impact of Cp on EGCG oxidation, human Cp and its anti-body protein was employed. EGCG was incubated in 0.15 M pH 7.4 PBS in the absence or presence of various forms of Cp or anti-Cp monitoring the formation of red EGCG oxidation products at OD_513nm_ and the remaining EGCG with HPLC. As shown in Figure 6A,B, Cp dose- and time- dependent enhanced EGCG oxidation were accompanied with reducing EGCG and increasing its oxidation products. However, the level of ROS, mainly contributed by superoxide anion and hydrogen peroxide, significantly decreased in EGCG oxidation with Cp (Figure 6C). Besides, the above phenomenon can be weakened after adding anti-Cp (Figure 6). Therefore, Cp can promotes EGCG oxidation accompanied with decreasing ROS levels.

## 3. Discussion

In the present study, we elucidated that diverse Cu levels can cure the high dose EGCG toxicity in animals. We found that Cu levels and serum Cp activity and hepatic NPFT level was significantly increased with a dose-dependent way in mice after Cu diet (1.68, 11.72 or 51.69 mg/kg) treatments; however, there was no impact on SOD activity. Interestingly, EGCG toxicity (750 mg/kg) was enhanced in Cu-deficient mice leading to severe death; however, all mice survived in Cu-super nutrition mice. The activities of ALT and AST significantly increased at 24 h after EGCG treatment in Cu-deficient mice, which was inhibited in Cu-super nutrition or -sufficient mice. Besides, Cp can promotes EGCG oxidation accompanied with decreasing levels in vitro. These results demonstrated that Cu status can mediated EGCG hepatotoxicity via the regulation on serum Cp activity and hepatic NPFT level, suggesting that the use of Cu together with EGCG promotes its applications.

The previous studies have demonstrated that a single administration of high dose of EGCG (750 and 1500 mg/kg, i.g.) caused hepatotoxicity and increased plasma ALT in mice [8]. While, multiple doses of EGCG (500 mg/kg, i.g.) once daily treated dosed to Beagle dogs caused injury to the liver, kidney and gastro intestinal tract toxicity [9]. Furthermore, the multiple doses of EGCG (100 mg/kg, i.p.) enhanced ALT levels in mice and EGCG (150 mg/kg, i.p.) within less than 24 h, induced death in animals [10]. However, the underlying protective factors against the hepatotoxicity of green tea extract or EGCG were not studied in these reports, only few of the researchers investigated these shield factors. Recently, [12] reported that melatonin can attenuate EGCG-triggered hepatotoxicity and reduce hepatic Nrf2 activation, indicating that there could be some factors that can influence EGCG hepatotoxicity. The results in this present study showed that a single dose of EGCG (750 mg/kg) increased plasma ALT and AST, which caused severe hepatotoxicity leading to serious and moderate deaths in Cu-deficient and Cu-sufficient mice respectively; however, Cu-super nutrition mice survived successfully, suggesting that EGCG-triggered hepatotoxicity associated with diverse Cu levels in mice.

Cu is readily absorbed from the stomach and small intestine. After nutritional requirements are met, there are several mechanisms that prevent Cu overload. Excess Cu absorbed into gastrointestinal mucosal cells induces the synthesis of metallothionein and binds to the metal binding protein metallothionein. This bound Cu is excreted when the cell is sloughed off. Cu that eludes binding to intestinal metallothionen is transported to the liver. It is stored in the liver bound to liver metallothionen, from which it is ultimately released into bile and excreted in the feces. Although Cu homeostasis plays an important role in the prevention of Cu toxicity, exposure to excessive levels of Cu can result in a number of adverse health effects including liver and kidney damage, anemia, immunotoxicity, and developmental toxicity. Many of these effects are consistent with oxidative damage to membranes or macromolecules. Excessive Cu causes DNA damage, lipid peroxidation and protein dysfunction [16,21]. Some literatures have reported that Cu overload have detrimental effects on hepatocellular structure [27] and mitochondrial function [28]. Generally, 4–13 mg/kg of Cu in diet is adequate in mice; and there is a supper nutrition diet with over 20 mg/kg of Cu [27,29,30,31]. There exists developmental toxicity after intake of more than 40 mg Cu kg^−1^·day^−1^ of Cu sulfate; while hepatic and renal toxicity occur with intake of more than 1000 mg Cu kg^−1^·day^−1^ of Cu sulfate [32,33]. In this present study, basal diet containing 1.68 mg/kg of Cu (no extra addition of Cu compounds) was labeled as Cu-deficient diet; whereas Cu-sufficient and Cu-super nutrition diets were supplemented with Cu of 10.0 and 50 mg/kg, respectively; and its Cu contents is 11.72 and 51.69 mg/kg detected by ICP-MS using copper sulfate as standard. It was not observed significant loael in mice after Cu-super nutrition diet in the study, in which the biomarkers of hepatic and renal toxicity including ALT, AST, BUN and Creatinine were no significant change among different Cu content diets (Figure 3A–D).

Cu-deficient mice developed anemia with low tissue Cu levels in liver or kidney and decreasing Cp activity in serum [14]. Moreover, serum Cp is a sensitive biomarker for Cu deficiency [19]. We found that serum Cp activities significantly increased in mice fed with Cu-super nutrition or -sufficient diet with a dose-dependent effect compared with mice fed with Cu-deficient diet and Cp mRNA level in liver significantly increased by 7.6- or 11.9-fold in mice fed with Cu-super nutrition diet or Cu-super nutrition diet plus EGCG respectively (Figure 5B). Serum Cp is also called serum polyphenol oxidase activity [34] which could participate in the EGCG oxidation in blood in mice. We found that although the dose of EGCG was not caused death in mice fed with Cu-super nutrition diet, it induced severe toxicity resulting in death in mice fed with Cu-deficient and Cu-sufficient diet, indicating that EGCG hepatotoxicity is associated with serum Cp activity in mice.

The toxicological actions of high dose of EGCG are considered to be due to its pro-oxidant properties generating ROS [8]. The data showed that hepatic NPFT, with GSH being a predominant component, significantly increased in mice fed with Cu-super nutrition or -sufficient diet with a dose-dependent effect compared with those fed with Cu-deficient diet; whereas the activity of other antioxidant enzymes including GR, GPx, GST, MDA and CAT were not significantly changed after drugs administration in liver of mice, indicating that EGCG hepatotoxicity is associated with hepatic GSH level in mice. Although SOD1 is an important cuproenzymes requiring Cu for full catalytic activity that catalyzes the dismutation of superoxide into molecular oxygen and hydrogen peroxide inactivating EGCG oxidation in vitro [35], the activity of SOD in serum and liver has no significant changes indicating that enhanced EGCG hepatotoxicity induced by Cu–deficiency is not involves SOD in mice.

Recent, Zhang et al. was reported that EGCG hepatotoxicity can be enhanced in mice treated with diethyldithiocarbamates, which can possibly lead to severe lethality [36]. They have demonstrated that co-administration of EGCG and diethyldithiocarbamate (DEDTC) both at tolerable levels caused lethality. It was found that the co-administration significantly increased hepatic Cu levels as well as decreased serum SOD activity caused by DEDTC. Moreover, the co-administration drastically increased lipid peroxidation, DNA damage and cell apoptosis as well as caused deleterious transcriptional responses including basal and Nrf2 antioxidant systems in the liver. The results suggest that DEDTC can act as a copper ionophore to increase hepatic levels of redox-active copper which promotes EGCG auto-oxidation to produce oxidative stress and toxicity. Similarly, it was reported that EGCG caused more extensive DNA degradation in copper-overload lymphocytes isolated from rats supplemented with copper compared to lymphocytes isolated from normal rats [37]. More, Supplementation with copper enhances anti-cancer action of EGCG in prostate cancer cell and involves mobilization of endogenous copper and ROS generation in vitro [38]. These studies demonstrated that the toxicity and cytotoxicity of EGCG was increased in Cu-overload tissues or cells. However, in the present study, it was observed that EGCG toxicity was protected in mice with supra-physiological levels of Cu involving modulation of serum Cp activity and ROS generation. Not only does administration of DEDTC or disulfiram, which is metabolized to DEDTC in vivo, significantly increase hepatic Cu levels [36,39,40,41], it also significantly decreases Cu levels or Cp activities in blood [36,42,43]. It is possible that because of the low Cu and Cp activities, more EGCG was translated into liver and oxidized with ROS generation resulting into hepatotoxicity in vivo.

In addition, supplementation of Cu decreased cytochrome P450 (CYP450) system activity in hepatocytes and cancer cells [44,45]. Moreover, the expanded active site pocket of some CYP enzymes, the promiscuous nature of the PXR ligand-binding pocket, as well as a marked affinity of some phytochemicals toward AhR, make CYP enzymes especially receptive to natural polyphenols [46]. However, there is no evidence that CYP is involved in EGCG metabolism [47]. Hence, the potential explanation that decreased CYP450 system activity in liver after supplementation of Cu lead to the low bioavailability and hepatotoxicity of EGCG need be further investigated.

In conclusion, we revealed that Cu had impacts on the toxicity of EGCG in mice. The present study, investigated that Cu-deficient and Cu-sufficient models showed increased severe and moderate hepatotoxicity in mice respectively. However, intake of Cu-super nutrition did not cause toxicity after EGCG administration. These results indicate that various dietary Cu levels can mediate EGCG hepatotoxicity via the regulation on serum Cp activity and hepatic NPFT levels. These findings suggest that, the use of Cu together with EGCG can promote its application. Although Cu supplement can reduce EGCG hepatotoxicity in mice, it is an important cognition that there is a different tolerance for copper between humans and mice—severe toxicity in humans occurs after a high dose of Cu. Therefore, the relationship of EGCG toxicity and the levels of Cu and cuprein in tissues should be further investigated. To our knowledge, there have been no published previous reports regarding the effect of dietary Cu on EGCG hepatotoxicity, which can be lethal. The observations in the present study, therefore, point an important element of green tea catechins which are being offered to the public in daily life. Thus, the result reported herein emphasizes the need for caution in the application of green tea catechins, and also gives occupational health scientists a new insight into green tea catechins, the potential of which is favorable or adverse interaction with Cu and Cu enzymes was heretofore unreported.

## 4. Materials and Methods

### 4.1. Ethical Guidelines

Animal care and protocols were in accordance with and approved by the Institutional Animals Ethics Committee of Nanjing Agricultural University and conformed to the Code of Practice for the Housing and Care of Animals Used in Scientific Procedures for the Use of Animals in Nutrition and Toxicity Research.

### 4.2. Chemicals and Drugs

Nicotinamide adenine dinucleotide phosphate (NADPH), reduced glutathione (GSH), oxidized glutathione (GSSG), 5,5′-dithiobis-(2-nitrobenzoic acid) (DTNB), 1-chloro-2,4-dinitrobenzene (CDNB) and glutathione reductase (GR, from Baker yeast) were purchased from Sigma (St. Louis, MO, USA). EGCG (99.8% purity) was purchased from Ebeikar Tea & Extracts Co. Ltd. (Hangzhou, China). All of these chemicals were of the highest grade available.

### 4.3. Animals Experiments

Male ICR-mice (8–10 g, 2–3 weeks old) were purchased from Changzhou Cavens Laboratory Animal Co. Ltd. (Changzhou, China). Diets containing different Cu levels were purchased from Jiangsu Medicience Co. Ltd. (Yangzhou, China). Their nutritional contents are shown in Table 1. The mice were housed in plastic cages in a room with controlled temperature (25 ± 1 °C), humidity (50 ± 10%) and 12 h light/dark cycle. Mice had access to food and water *ad libitum*. 

To establish the Cu-deficient, -sufficient, and -super nutrition mice models, ninety mice were randomly divided into three groups with thirty mice per group. Each group was fed with one of three diets containing Cu-deficient (1.68 mg/kg), Cu-sufficient (11.72 mg/kg), and Cu-super nutrition (51.69 mg/kg) Cu, respectively, for twenty-eight consecutive days. Mice were weighed daily.

To investigate the impact of dietary Cu levels on EGCG toxicity, mice from each model were randomly divided into two equal groups. The two groups were administered with either saline as control or EGCG at a single dose of 750 mg/kg by gavages. Mice were sacrificed at day 1 or after EGCG treatment, respectively. Fourteen mice (saline- and 7 EGCG-treated mice) were sacrificed at day 1 for evaluation of the acute EGCG toxicity. The remaining 16 mice/model (8 saline- and 8 EGCG-treated mice) were kept for 14 days to assess the effect of EGCG on animal survival. Mice were monitored daily.

### 4.4. Tissue Preparation and Biomarkers Assessment

Mice were sacrificed by cervical dislocation. Peripheral blood was obtained by retro orbital sinus bleeding. Serum biomarkers were determined using commercially available kits. The levels of alanine transaminase (ALT), aspartate transaminase (AST), blood urea nitrogen (BUN), ceruloplasmin (Cp) and creatinine (Cr) in serum were determined using kits purchased from the Nanjing Jiancheng Bioengineering Institute (Nanjing, China).

Hepatic tissues were excised, rinsed in ice-cold saline, and then stored at −30 °C before assay. Liver samples were homogenized in ice-cold 150 mM pH 7.2 phosphate buffer solution (PBS) containing 1 mM EDTANa_2_. The homogenate was centrifuged at 15,000× *g* at 4 °C for 15 min. Protein levels in the resultant supernatant were determined by the Bradford dye-binding assay with bovine serum albumin as a standard [48].

For measurement of non-protein free thiol (NPFT) levels, with GSH being a predominant component, an aliquot of homogenate was taken out immediately after homogenization and mixed with trichloroacetic acid (20%, *w*/*v*), at the volume ratio of 9:1. This procedure permits precipitation of all proteins in the homogenate and make GSH in the homogenate stable at 4 °C for at least 2 h. The trichloroacetic acid-treated homogenate was centrifuged at 10,000× *g* at 4 °C for 5 min. Within 2 h after centrifugation, the supernatant was mixed with DTNB (20 mg/mL in 0.2 MPBS, pH 8.0) and absorbance was read at 412 nm. NPFT level was expressed as nmol GSH/mg protein [49].

Malondialdehyde (MDA) level in liver was determined using a commercial kit, which was also purchased from the Nanjing Jiancheng Bioengineering Institute (Nanjing, China). MDA level was expressed as nmol/mg protein.

Cu levels in liver were determined by using Inductively Coupled Plasma Mass Spectrometry (ICP-MS). The signal intensity recorded by ICP-MS was used to calculate Cu content with copper sulfate as standard.

Glutathione reductase (GR) and glutathione peroxidase (GPx) activities were assessed as described by [50,51], respectively. GR activity was calculated in terms of NADPH oxidized nmols/min/mg protein. GPx activity was expressed as NADPH oxidized µmol/min/mg protein. Glutathione S-transferase (GST) activity was determined by using CDNB [52]. One unit of GST activity was calculated in terms of nmols CDNB changed/min/mg protein. Superoxide dismutase (SOD) activity was estimated with the xanthine/xanthine oxidase and nitroblue tetrazolium system. One unit of SOD activity was defined as the amount of protein that inhibits the rate of nitroblue tetrazolium reduction by 50% [53]. Catalase (CAT) activity was assayed on the basis of its ability to decompose H_2_O_2_ that was measured at 240 nm [54]. One unit of CAT activity was defined in terms of nmol H_2_O_2_ consumed/min/mg protein. Data are expressed as U/mg protein.

### 4.5. RNA Isolation, Reverse Transcriptase Polymerase Chain Reaction and Real-Time PCR

Total RNA was isolated using TRIzol reagent according to the manufacturer’s protocol (Takara Biotechnology, Shiga, Japan). RNA samples having a ratio of A260 nm:A280 nm more than 1.8 were used for RT-PCR. cDNA was generated using 50 ng of total RNA, oligodT primer, and PrimeScript RT Enzyme Mix according to the manufacturer’s instructions (RT-qPCR kit, Takara Biotechnology) in a total volume of 20 μL. Real-time PCR was performed with a CFX System (BioRad, Berkeley, CA, USA) according to the manufacturer’s protocol (Takara Biotechnology). ΔCT values were determined by normalizing to β-actin. Fold changes were calculated using 2^−(ΔΔ*C*T)^. The gene-specific primers as shown in Table 2 were designed using available gene sequences.

### 4.6. EGCG Oxidation Assessment

EGCG was incubated in 0.15 M pH 7.4 PBS at 25 °C in the absence or presence of various forms of Cp (No: C4519, Sigma, St. Louis, MO, USA) or anti-Cp (No: HPA001834, Sigma, St. Louis, MO, USA). Along with the EGCG oxidation, the EGCG solution gradually developed red color, thus absorbance at 513 nm was used for assessing EGCG oxidation. Since Cp could enhance EGCG oxidation, accordingly, the values of OD_513nm_ increased. Thus, this property was used for comparing pro-oxidant effect of Cp on EGCG oxidation. Kinetic alterations of OD_513nm_ were recorded using a plate reader (Cytation3, BioTek, Winooski, VT, USA). For quantitating EGCG in the above systems, high performance liquid chromatography (HPLC, Alliance 2695, Waters, Milford, MA, USA) was employed according to a standard method (GB/T 8313-2008). Briefly, chromatographic separation was performed on a C18 reversed-phase column (250 × 4.60 mm, 5 μm particle size, Waters, Milford, MA, USA). The mobile phase consisted of (A) deionized water with 9.0% acetonitrile and 0.2% acetic acid; and (B) 80.0% acetonitrile and 0.2% acetic acid. The following gradients were applied according to the following procedures: 100% A, 0–10 min; a linear gradient 68% A, 11–25 min; 68% A, 26–35 min; and a linear gradient up to 100% A, 35–45 min. The column temperature was set at 25 °C. The injection volume of samples was 5 μL. The elution rate was 1 mL/min, and the detection wavelength was set at 278 nm.

### 4.7. ROS Detection

To detect ROS levels produced by EGCG in the absence or presence of various forms of Cp or Anti-Cp, EGCG and compounds containing Cu were incubated in 0.15 M pH 7.4 PBS with 50 μM DCFH-DA as a probe of fluorescence at 37 °C. Fluorescence was excited at 488 nm and kinetic alterations of fluorescence intensity were recorded at 525 nm using a plate reader (Cytation3, BioTek, Winooski, VT, USA).

### 4.8. Statistical Analyses

All data were presented as Means ± SEM. The differences between groups were evaluated by analysis of variance (ANOVA) test or the Student’s *t* test. The Kaplan-Meier method was used to evaluate survival, and the differences were analyzed by the Log-rank test. All *p*-values were two-sided and results were considered statistically significant at *p* values < 0.05.

## Figures and Tables

**Table 1:** The contents of nutrition in copper diets per kilogram.

	Copper-Deficient Diet	Copper-Sufficient Diet	Copper-Super Nutrition Diet
Casein (g)	200.0	200.0	200.0
l-cystine (g)	30.0	30.0	30.0
Corn Starch (g)	397.5	397.5	397.5
Maltodextrin (g)	132.0	132.0	132.0
Sucrose (g)	100.0	100.0	100.0
Cellulose (g)	50.0	50.0	50.0
Soybean Oil (g)	70.0	70.0	70.0
Choline Bitartrate (g)	2.5	2.5	2.5
Vitamin Complex (g)	10.0	10.0	10.0
Mine Complex (g)	35.0	35.0	35.0
Ca (g)	5.0	5.0	5.0
P (g)	3.0	3.0	3.0
K (g)	3.6	3.6	3.6
S (g)	0.3	0.3	0.3
Mg (g)	0.5	0.5	0.5
Na (g)	1.0	1.0	1.0
Cl (g)	1.6	1.6	1.6
Cu (mg) *	0.0	10.0	50.0
Fe (mg)	45.0	45.0	45.0
Mn (mg)	10.2	10.2	10.2
Zn (mg)	30.0	30.0	30.0
F (mg)	1.0	1.0	1.0
Se (mg)	0.2	0.2	0.2
I (mg)	0.2	0.2	0.2

* Cu contents in diet is 1.68, 11.72 and 51.69 mg/kg, respectively, detected by ICP-MS using copper sulfate as standard.

**Table 2:** Gene-specific primers used.

Genes	Primers	Sequences
SOD1	Sense	TGGAGACCTGGGCAATGTGACT
	Antisense	TCCACCTTTGCCCAAGTCATCT
Cp1	Sense	TTCAGTGCCAGAAGCATAGTCCCA
	Antisense	GGCCACAGGGAACTGTGTTTGTTT
β-Actin	Sense	GCTGAGAGGGAAATCGTGCGT
	Antisense	ACCGCTCGTTGCCAATAGTGA
